# The Intellectual Landscape of Nanovaccines: A Bibliometric Perspective on Scientific Progress and Future Directions

**DOI:** 10.7759/cureus.60131

**Published:** 2024-05-12

**Authors:** Jobin Jose, Vinoj M N, Bindu R G, Anat Suman Jose, Jacob Jubin

**Affiliations:** 1 Library, Marian College Kuttikkanam (Autonomous), Kuttikkanam, IND; 2 Physics, St. Peter's College, Kolenchery, Kolenchery, IND; 3 Physics, Nair Service Society (NSS) College, Nilamel, Kollam, IND; 4 Library, St. Peter's College, Kolenchery, Kolenchery, IND; 5 Library, St. Thomas College, Palai (Autonomous), Palai, IND

**Keywords:** nano-vaccine, citespace, biblioshiny, biblimetric analysis, nanovaccine, nano vaccine

## Abstract

This bibliometric study provides a comprehensive analysis of the burgeoning field of nanovaccine research, leveraging data sourced from Scopus and employing the Preferred Reporting Items for Systematic reviews and Meta-Analyses (PRISMA) flowchart for the meticulous screening, inclusion, and exclusion of relevant studies. Utilizing sophisticated bibliometric tools, such as Biblioshiny and CiteSpace, we dissected the expansive literature to unearth critical insights into the annual scientific output, identifying key contributors and pivotal publications that have shaped the domain. The analysis delineates the most influential authors, sources, and globally cited documents, offering a macroscopic view of the field’s intellectual structure and growth trajectory. Trend topics and thematic mapping underscored the evolution of research foci, from fundamental immunological mechanisms to cutting-edge nanomaterial applications. Factorial analysis and keyword co-occurrence networks revealed the intricate associations and thematic concentrations within the literature. The study’s robust methodology also pinpointed the keywords exhibiting the strongest citation bursts, signifying emergent areas of intense academic interest. Networks of cited authors illuminated collaborative patterns among scholars, while timeline network visualizations of country collaborations depicted the global interplay in nanovaccine development. Crucially, this study identified notable research gaps and practical implications, suggesting directions for future investigation and highlighting the translational potential of nanovaccines in public health and personalized medicine. This bibliometric investigation not only maps the current landscape but also charts a course for the trajectory of nanovaccine research, emphasizing its role as a cornerstone of innovative immunotherapeutic strategies.

## Introduction and background

Nanovaccines represent a pivotal advancement in the field of immunization, utilizing the novel properties of nanoscale materials to enhance vaccine efficacy, safety, and delivery [1-4]. By engineering particles at the nanometer scale, akin to the size of viruses and small molecules, scientists can precisely target and stimulate the immune system in unprecedented ways [5-7]. This approach promises to revolutionize how vaccines are designed, enabling the development of more potent and specific responses against infectious diseases, cancers, and autoimmune disorders [8,9].

The central breakaway of nanovaccines is based on their ability to design pathogens’ size and shape, creating a more realistic playing field for the immune system [2,10]. Biomimicry achieves this and helps the body when exposed to actual threats [11-13]. In addition, nanomaterials can absorb antigens and adjuvants, protecting them from decomposition and enabling them to be released in a controlled manner; this enhances immunogenicity while minimizing side effects [14,15]. Nanovaccines can enable tumor vaccination using needle-free delivery methods such as nasal sprays and oral vaccines, which is a significant advancement [16]. It is beneficial to usage and administration; it also reduces the incidence of needle sewing and possible contamination [17,18]. Nanovaccine is structurally and biologically stable and has the potential to produce formulations that do not need to be stored in the cold chain. It may improve the feasibility of vaccine distribution in an underserved field [4].

However, as promising as these features may seem, the development of effective nanovaccines is encumbered by the necessity of conducting safety studies and the prospects for scalability [6]. Consequently, as the research on the subject continues, increased collaboration between the realms of nanotechnology, immunology, and pharmacology is going to be required [19,20]. Thus, the potential of nanovaccines to protect individuals from multiple conditions will be fully tapped.

Bibliometric analysis is one of the most common approaches applied to scientific research, enabling a quantitative assessment of the literature in different scientific areas, and nanovaccine research is no exception [21-24]. The use of tools such as Biblioshiny and CiteSpace allows exploration of the field’s dynamics, current trends, and prospects. Moreover, their application is especially meaningful in exploring innovations and eligible technologies, ensuring research continuity and confidence concerning its outcomes. By analyzing large datasets related to publications, the researchers may determine publication dynamics, citation behavior, co-authorship, and clusters, as well as conduct mapping of the field and its intellectual and collaborative sides, contributors, and beneficiaries [25-27].

As a result, combining CiteSpace and Biblioshiny, the user-friendly web interface of the Bibliometrix R package, would allow for conducting a comprehensive bibliometric analysis while improving the visualization of data and enhancing its interpretation [28-31]. While Biblioshiny’s bibliometric data may be detailed and cover the growth in the literature, distribution among journals, and frequently cited articles regarding nanovaccine, CiteSpace, in turn, would support the user in identifying and visualizing the trends, shifts in paradigms, and key publications, authors, and journals [32-34]. Additionally, this tool identifies “burst” terms that may be interpreted as an indication of increased interest, which may support the researcher in anticipating future trends in research. Therefore, both the CiteSpace and Biblioshiny tools could be used to navigate the complexity of the nanovaccine field and address the challenges in the healthcare sector.

This study seeks to address the following research questions: (RQ1) What are the core research themes and emerging trends in nanovaccine research?; (RQ2) Who are the key contributors, and what are their collaborative networks in the field of nanovaccine research?; (RQ3) How has the publication output in nanovaccine research evolved over time?; (RQ4) What are the most influential publications in nanovaccine research?; and (RQ5) What are the existing research gaps within nanovaccine?

Materials and methods

**Figure 1:**
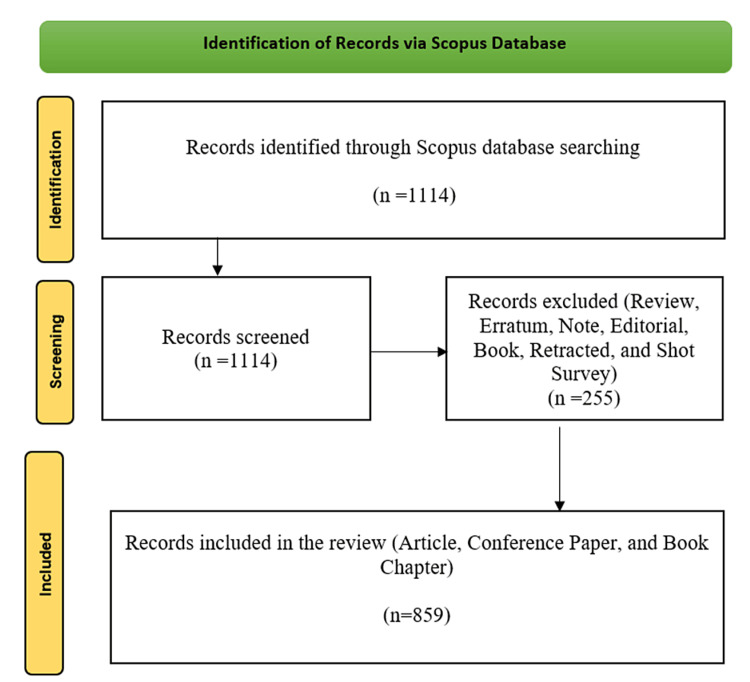
PRISMA flow diagram used to identify, screen, and include papers in the bibliometric analysis PRISMA, Preferred Reporting Items for Systematic reviews and Meta-Analyses

Scopus was chosen as this study's primary bibliographical data source because it covers a broader range of quality journals compared to other databases [35-37]. The records were retrieved using the query “( TITLE-ABS-KEY ( nanovaccine ) OR TITLE-ABS-KEY ( nano-vaccine ) OR TITLE-ABS-KEY ( "nano vaccine" ) )”. There were no language restrictions; only journal articles, conference papers, and book chapters were considered. A total of 859 documents have been gathered from 280 distinct sources between 2004 and 2024. Figure 1 illustrates the Preferred Reporting Items for Systematic reviews and Meta-Analyses (PRISMA) approach to selecting papers for bibliometric analysis. It is a three-phase procedure in which we identify and extract the data for analysis initially from the databases. We excluded reviews, editorials, books, short notes, and surveys in the second phase. Documents included are articles, conference papers, and book chapters. The findings were stored as “CSV” and RIS files, and bibliometric analysis was performed on the data using CiteSpace version 6.2.R3 (Advanced) and Biblioshiny software. The main aspects of this investigation are summarized in Table 1.

**Table 1:** Key elements of the investigation

Description	Results
Main information about the data	
Time span	2004:2024
Sources (journals, books, etc.)	280
Documents	859
Annual growth rate, %	23.84
Document average age	3.44
Average citations per doc	22.4
References	41,471
Document contents	
Keywords plus (ID)	6,789
Author’s keywords (DE)	2,072
Authors	
Authors	3,377
Authors of single-authored docs	5
Authors collaboration	
Single-authored docs	5
Co-authors per doc	8.41
International co-authorships, %	22
Document types	
Article	811
Book chapter	32
Conference paper	16

## Review

Findings and discussions

Annual Scientific Production

**Figure 2:**
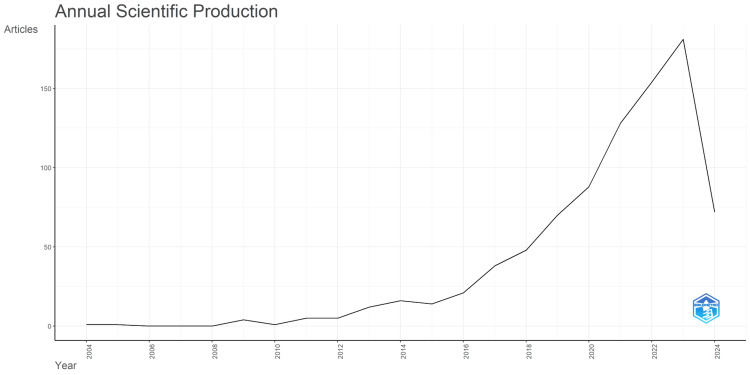
Yearly production of scientific articles from 2004 to 2024

Figure 2, showcasing annual scientific production from 2004 to 2024, reveals a dynamic growth in nanovaccine research, initially characterized by limited output from 2004 to 2008, suggesting the field was in its early stages. This is followed by a gradual increase in publications from 2009 to 2012, indicating rising interest and possible breakthroughs in nanovaccine technology. Between 2013 and 2017, a consistent and significant rise in scientific articles signaled the growing recognition of nanovaccines’ potential. The period from 2018 to 2023 is marked by a robust expansion, reflecting the field’s ascent to a major area of scientific endeavor, likely propelled by advancements and the increasing applicability of nanotechnology in vaccine development. The count for 2024, at 72 articles, although only partway through the year, suggests that the upward trajectory in nanovaccine research is set to continue, underpinning the field’s critical role in advancing vaccine science.

Documents by Authors

Table 2 shows key contributors to nanovaccine research, with Narasimhan leading the pack with 34 documents, underscoring his prominence in the field. Wannemuehler, with 17 publications, and Kong, with 15, also stand out, likely focusing on specialized aspects of nanovaccine technology and novel delivery systems, respectively. Ross and Toth, each with 12 documents, as well as Liu with 11, are pivotal in shaping research on vaccine formulation and efficacy. Dennis, Hu, Renu, and Renukaradhya, all with 10 publications, contribute to a broad range of topics that support the expansive dialogue on nanovaccines. This array of authors and their contributions illustrates a dynamic, diverse research community deeply engaged in advancing nanovaccine development and application, reflecting a robust collective effort to enhance medical science through innovative vaccine technologies.

**Table 2:** Documents by authors

Author name	Number of documents
Narasimhan	34
Wannemuehler	17
Kong	15
Ross	12
Toth	12
Liu	11
Dennis	10
Hu	10
Renu	10
Renukaradhya	10

Network Visualization of Co-Citation of Authors

**Figure 3:**
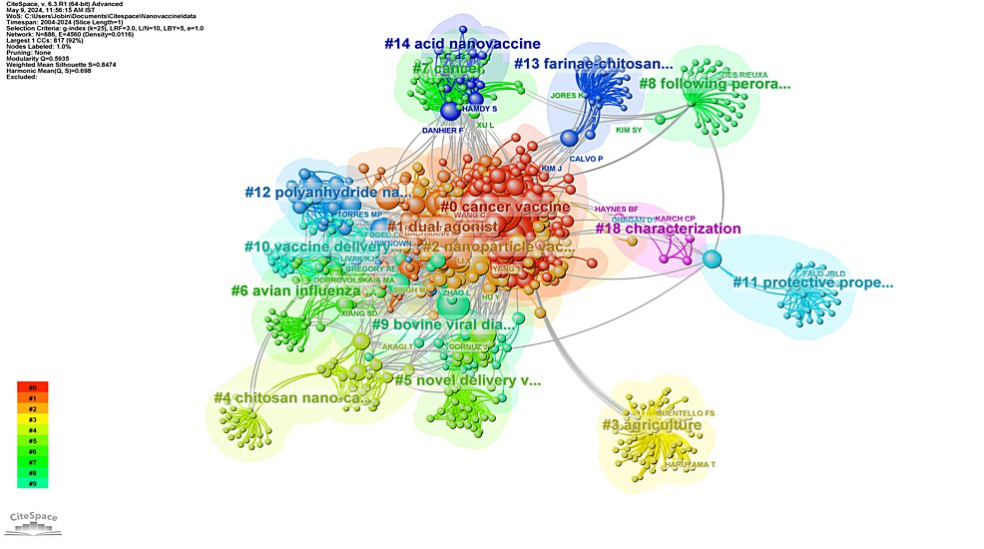
Network visualization of co-citation of authors

Figure 3 depicts the network visualization of co-citation among authors in the field of nanovaccine research, showing a complex landscape with 16 distinct clusters, each representing a unique subdomain within the broader research area. Cluster #0, the “Cancer Vaccine” cluster, stands out with 220 members, where Zhang’s 2023 work on cancer therapy using nanoadjuvants is highly cited, especially by members Zhang, Wang, and Xu. In Cluster #1, “Dual Agonist,” comprising 100 members, the primary reference is Correia-Pinto’s 2013 vaccine carrier study, with Bachmann, Reddy, and Li as key figures. Cluster #2’s 84 “Nanoparticle Vaccine” researchers center around Zhao’s 2023 paper on tumor immunity, citing Li, Yang, and Skwarczynski. Cluster #3, focused on “Agriculture” with 66 members, references Shrivastava’s 2009 agrifood nanotechnology work, while Cluster #4 and Cluster #5, having 44 and 41 members, respectively, spotlight Zheng’s 2014 research on novel nanocarriers for vaccines. Shan’s 2010 study on avian influenza vaccines highlights Cluster #6’s 40 members and Cluster #7’s 39 “Cancer” researchers cite Shi’s 2005 paper. Ge’s 2009 study on the peroral vaccine route characterizes Cluster #8 with 36 members. Cluster #9, containing 31 members, focuses on bovine viral diarrhea virus research, featuring Mahony’s 2015 study. Vaccine delivery is the theme for Cluster #10’s 30 members, with Correia-Pinto’s 2013 article providing insight. Cluster #11, with 24 members, and Cluster #12, with 22 members, are notable for research on protective properties and polyanhydride nanovaccines, respectively. Cluster #13, “Farinae-Chitosan Vaccine,” with 21 members, revolves around Liu’s 2009 nasal immunotherapy study, whereas Cluster #14, “Acid Nanovaccine,” with 14 members, cites Athanasiou’s 2017 work. Lastly, Cluster #18, “Characterization,” although the smallest with five members, features Karch’s 2019 research on vaccine-relevant protein nanoparticles. Each cluster, marked by a silhouette value of 0, hints at tightly interwoven research themes across the field.

Most Globally Cited Documents

Table 3 presents the most globally cited documents in nanovaccine research, providing insights into the impact and influence of specific works in the field. Luo’s 2017 publication in Nature Nanotechnology leads with a total of 620 citations and an impressive average of 77.5 citations per year, reflecting a high impact in the field. Fifis’ 2004 article in The Journal of Immunology also shows a significant number of total citations (576), but with a lower average of 27.43 citations per year, indicating enduring relevance over a longer period. Yang’s work in ACS Nano from 2018 quickly accumulated citations with a total of 497, suggesting rapid recognition of its contribution to nanovaccine research. Publications in Advanced Materials and ACS Nano by Kroll and Guo, respectively, also stand out for their citation performance, indicating these papers’ importance in advancing materials science within the context of nanovaccines. Furthermore, Xu’s paper published in Nature Nanotechnology in 2020 demonstrates a high citation rate, which is remarkable given the recency of the publication and highlights its potential importance in the future. Finally, Zhu’s and Liu’s works from ACS Nano and Molecular Therapy maintain a similar citation rate, illustrating the established importance of these papers. In conclusion, citation rates for Shi’s paper in Biomaterials in 2017 and Ding’s article in Angewandte Chemie International Edition in 2020 are solid, indicating high demand for the works in the academic community.

**Table 3:** Most globally cited documents

Paper	DOI	Total citations	Total citations per year	Normalized total citations
Luo (2017) [38], Nature Nanotechnology	10.1038/nnano.2017.52	620	77.5	9.56
Fifis (2004) [39], Journal of Immunology	10.4049/jimmunol.173.5.3148	576	27.43	1
Yang (2018) [40], ACS Nano	10.1021/acsnano.7b09041	497	71	10.16
Kroll (2017) [41], Advanced Materials	10.1002/adma.201703969	366	45.75	5.64
Guo (2015) [42], ACS Nano	10.1021/acsnano.5b01042	325	32.5	5.46
Xu (2020) [43], Nature Nanotechnology	10.1038/s41565-020-00781-4	315	63	8.24
Zhu (2017) [44], ACS Nano	10.1021/acsnano.7b00978	266	33.25	4.1
Liu (2018) [45], Molecular Therapy	10.1016/j.ymthe.2017.10.020	235	33.57	4.8
Shi (2017) [46], Biomaterials	10.1016/j.biomaterials.2016.10.047	219	27.38	3.38
Ding (2020) [47], Angewandte Chemie International Edition	10.1002/anie.202005111	218	43.6	5.7

Most Relevant Sources

Table 4 presents a list of journals keen on research in the nanovaccine field, with each implementing a unique factor arising from the field’s multidisciplinary nature. ACS Nano, complementing 42 articles, leads in researching new nanovaccine technologies, a goal dependent on cutting-edge nanoscience. Biomaterials and Frontiers in Immunology, publishing 37 and 34 articles, researched vaccine delivery materials and new immunological agents. Journal of Controlled Release and Nano Letters, releasing 25 and 23 articles, lead in the research of nanovaccine release mechanisms and molecular engineering. Advanced Healthcare Materials and International Journal of Nanomedicine, which are also releasing 22 articles, conduct research in unique application areas within healthcare. Vaccines and Advanced Science topics are broad-based vaccine-related, with 20 and 19 articles. Small, with 16 articles, maintains the gap in research both in nano- and micro-level research for medical purposes. The above journals complement a novel approach to both research methodologies in the mentioned areas as well as toward the co-development of scientific and practical healthcare solutions.

**Table 4:** Most relevant sources

Sources	Articles
ACS Nano	42
Biomaterials	37
Frontiers in Immunology	33
Journal of Controlled Release	25
Nano Letters	23
Advanced Healthcare Materials	22
International Journal of Nanomedicine	22
Advanced Science	19
Vaccines	19
Small	16

Countries’ Scientific Production

Table 5 shows the ranking of countries by the number of documents contributed. China is the forerunner with its impressive 438 documents, which is a testament to the extreme dedication and productivity on its part. The United States is next with 191 documents, meaning its contribution is just as high but not as dedicated as that of China. Iran clearly stands out, having contributed 54 works, which is more than India with its 48 documents, despite the massive population and scientifically popular research. Australia has made 38 contributions, the United Kingdom 30, Germany 26, Spain 24, South Korea 22, and the Netherlands 16. This categorization testifies to the global nature of nanovaccine research, with humanity focused on the issue from virtually all corners of the world. The significant lead of China also demonstrates that they invest in nanotechnology as a possible cornerstone of their successful vaccine development.

**Table 5:** Countries’ scientific production

Country/territory	Number of documents
China	439
United States	191
Iran	54
India	48
Australia	38
United Kingdom	30
Germany	26
Spain	24
South Korea	22
Netherlands	16

Timeline Network Visualization of Countries’ Collaborations

**Figure 4:**
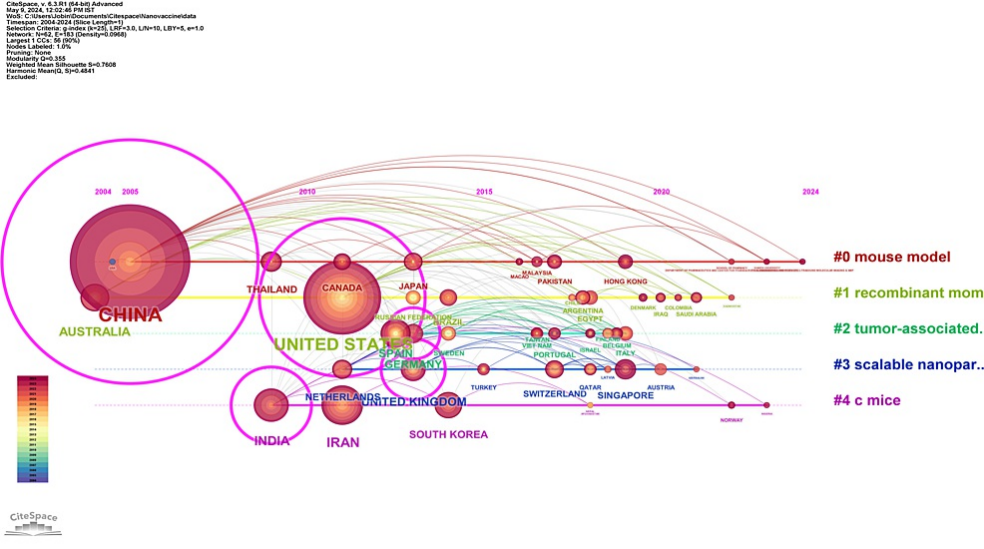
Timeline network visualization of countries’ collaborations

The CiteSpace visualization presented in Figure 4 demonstrates extensive worldwide collaboration in the field of nanovaccine research, which is composed of six distinct and focused clusters. Cluster #0, represented by 434 citations from China, once conducted the most studies of the mouse models critical for immunotherapy development, as evidenced by Su. Alto, Cluster #1 investigates the most mouse models, enhanced by CRISPR engineering and including contributions from Saudi Arabia, Kazakhstan, and Colombia, as demonstrated by Pappalardo. Furthermore, Cluster #2 includes Germany, Sweden, and Vietnam, which investigate research into tumor-associated macrophages, which is emphasized by Dölen in PLGA nanoparticles. The most cohesive cluster, Cluster #3, spotlights scalable nanoparticle vaccines, pivotal in the response to MERS coronavirus, with key input from the United Kingdom, the Netherlands, and Singapore, according to Mohsen’s 2021 study. Cluster #4 dives into cancer immunotherapy through “C mice” models, as illustrated by Rajendrakumar’s 2018 research, with notable citations from South Korea and India. Lastly, Cluster #5, although the smallest, shows dedicated research into oral nanovaccines, particularly Leroux’s 2023 publication on Echinococcus granulosus, with Uruguay, the Czech Republic, and Tunisia contributing. This map of intellectual synergy reveals not only a diverse array of research themes but also a dynamic evolution of partnerships, underscoring the international community’s enduring commitment to advancing vaccine technology.

Trend Topics

**Figure 5:**
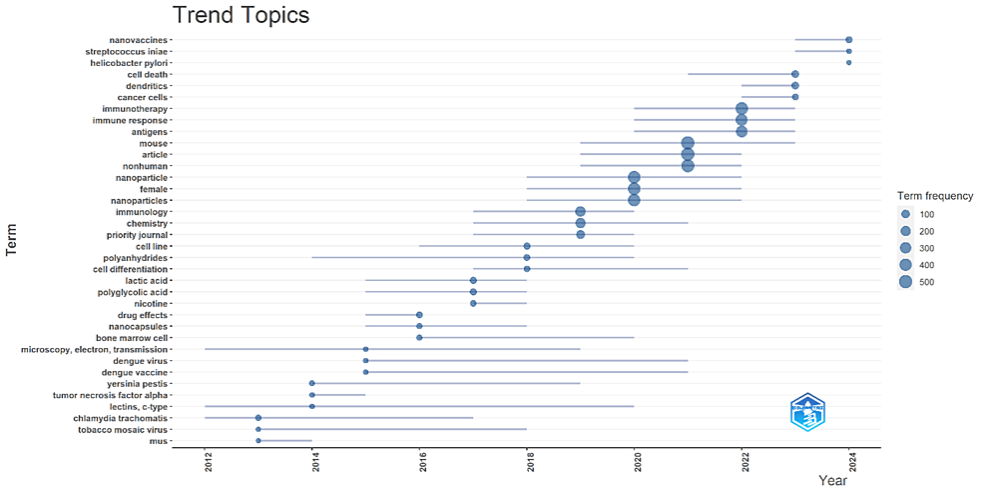
A visual indicating the popularity of topics

Figure 5 displays trend topics for nanovaccine research, showcasing the changing focus areas of researchers in the field. As can be seen, “nanovaccines,” “immunotherapy,” and “immune response” are key terms during the entire period and are always at the center of the visualization, which aligns with the field’s core topics. “Streptococcus iniae” and “Helicobacter pylori” are pathogens to which nanovaccines are applied specifically. “Dendritic cells,” “cancer cells,” and “antigens” are highly developed topics, which indicates that cancer immunotherapy and research on the mechanisms for immune activation are among the most actively studied issues. “Polyanhydrides,” “lactic acid,” and “polyglycolic acid” are terms that frame a biodegradable polymer to which great attention is paid in nanovaccine agents. One can note a frequent use of the terms “nanoparticle” and “nanocapsules” related to the issues of drug delivery and nanotechnology in general. “Dengue virus,” “Yersinia pestis,” and “dengue vaccine” are more recent topics, which shows that new infectious agents are becoming an increasing threat, and nanovaccine methods are applied to them. Moreover, “tumor necrosis factor alpha” and “lectins, c-type” can be a category of key molecules studied for their role in the immunological processes of vaccination. Thus, the visualization presents a dynamic research field interested in both the basic and the most advanced niche aspects of vaccination technology.

Thematic Map

**Figure 6:**
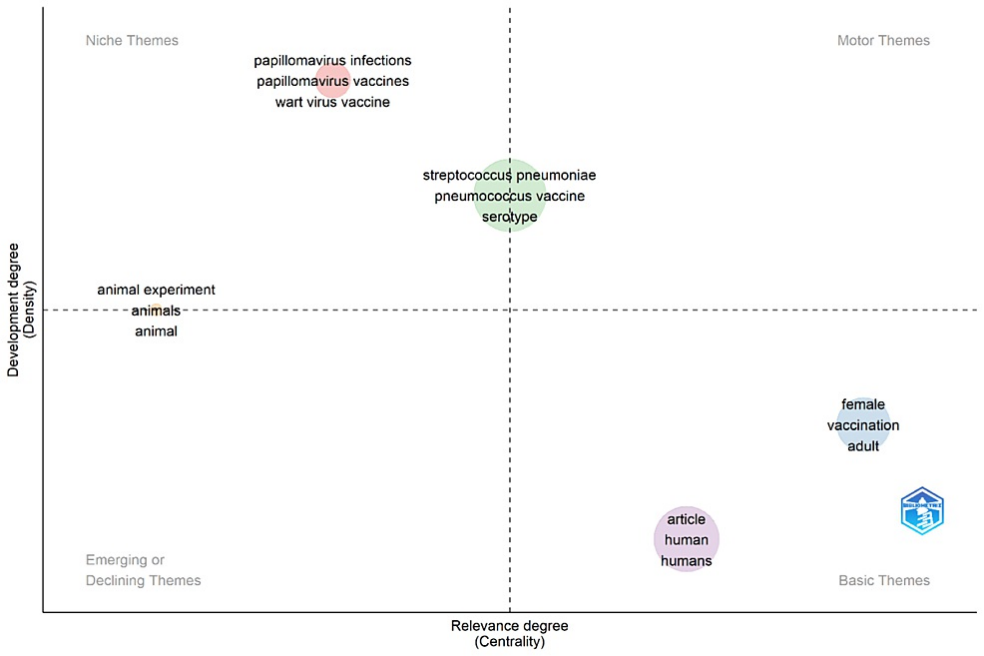
Thematic representation of keywords

The thematic map displayed in Figure 6 visualizes different areas of focus within nanovaccine research, classified by their niche, motor, basic, and emerging or declining status, based on two dimensions: development degree (density) and relevance degree (centrality) [48].

Niche themes: These are specialized areas with lower centrality and density, indicating they are not widely researched or central to the field but could represent cutting-edge or very specific areas of study. Here, we have topics like “papillomavirus infections,” “papillomavirus vaccines,” and “wart virus vaccine.” They may be highly specialized topics within nanovaccine research with a narrow scope of study.

Motor themes: These themes are high in both centrality and density, meaning they are well-developed and core to the field. In this case, “Streptococcus pneumoniae,” “pneumococcus vaccine,” and “serotype” are likely to be key drivers of research in the field, receiving a lot of attention and possibly funding.

Basic themes: Situated in the bottom right, these are foundational to the field with high centrality but lower density. They are central to the understanding of the field but may not be the current focus of active research. “Female,” “vaccination,” “adult,” “article,” “human,” and “humans” fall into this category, indicating that while these topics are foundational to nanovaccine research, they might not be at the forefront of current scientific inquiry.

Emerging or declining themes: These topics have low centrality and are on the cusp between low and medium density, suggesting they are either on the rise as new areas of interest or declining as the field moves on to other topics. “Animal experiment,” “animals,” and “animal” are grouped here, indicating that research on nanovaccines related to animal studies might be in a state of flux.

Conceptual Structure Map Using Multiple Correspondence Analysis

**Figure 7:**
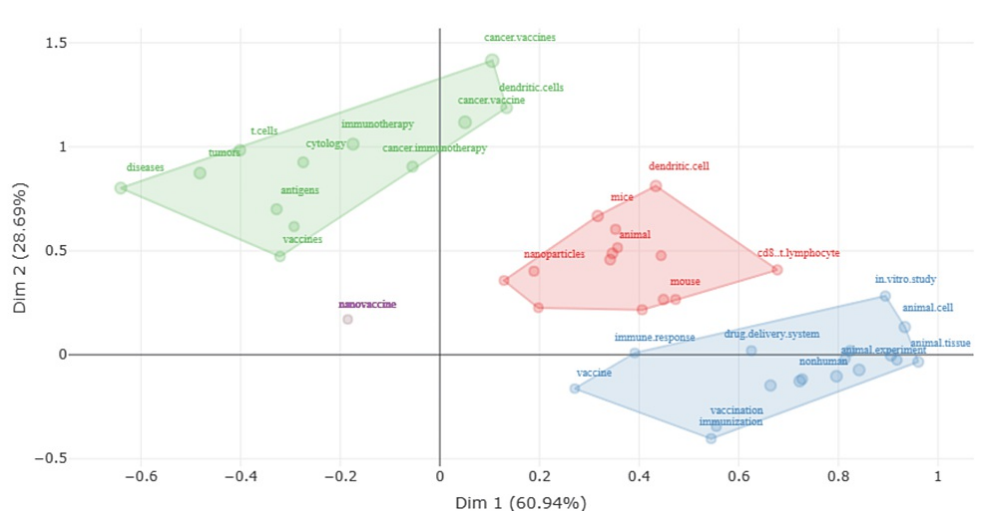
Conceptual structure map using multiple correspondence analysis

The factorial analysis is visual in Figure 7, which maps research themes in nanovaccine research to two principal dimensions, which account for a significant proportion of the variance in the dataset [49]. The first dimension (Dim 1), capturing over 61% of the variance, could represent the technical versus biological focus within the research, while the second dimension (Dim 2), explaining about 24%, might differentiate between the theoretical versus applied nature of the studies.

In the green quadrant, which correlates positively with both dimensions, we see terms like “cancer,” “vaccines,” “t cells,” “tumor,” and “immunotherapy.” This suggests a cluster of research that’s highly developed and likely focuses on the application of nanovaccines in cancer therapy, possibly exploring the immunogenicity and therapeutic potential of nanovaccines against tumors.

The red quadrant, with a high Dim 1 and low Dim 2 score, includes “dendritic cells,” “cd8 t lymphocyte,” and “in vitro study,” indicating a focus on the fundamental immune responses at the cellular level and controlled experimental studies in a laboratory setting.

Conversely, the blue quadrant, with a negative score on Dim 1 and a positive score on Dim 2, features “nanovaccine,” “immune response,” “vaccine,” “animal cell,” “animal tissue,” and “in vivo.” These terms may represent more applied research focused on the development and testing of nanovaccines in animal models.

Lastly, the purple dot representing “nanovaccine” at its origin suggests it is a central term in the field that may bridge both technical and biological aspects as well as theoretical and applied research within the context of nanovaccine studies.

Timeline Network Visualization of Co-occurrence of Keywords

**Figure 8:**
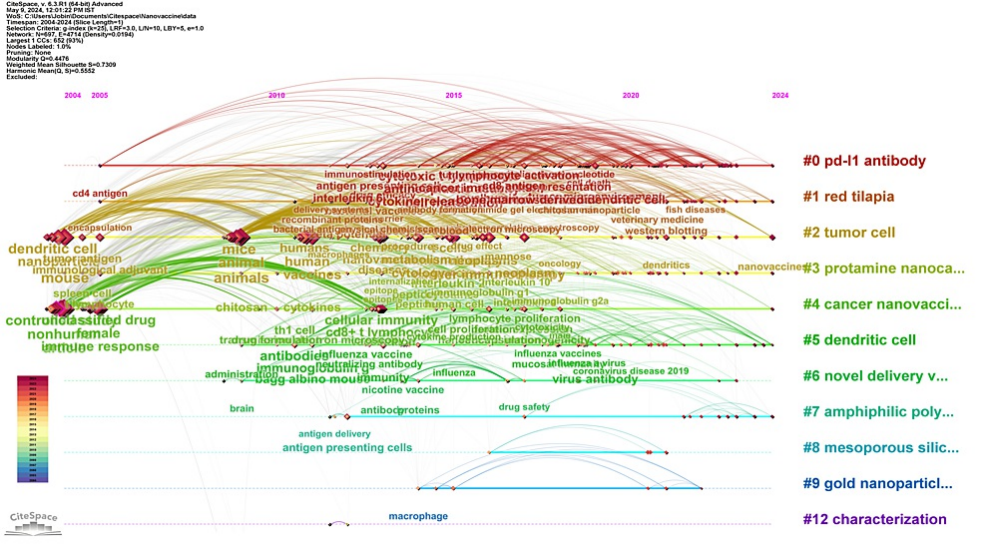
Co-occurrence network map of all keywords

Figure 8 shows a visualization of the multifaceted landscape of nanovaccine research. The visualization categorizes the research into 12 clusters based on keyword co-occurrence. Each cluster highlights distinct research foci and evolution patterns. Cluster #0, “PD-L1 Antibody,” the largest group with 157 members, delves into immunotherapy, particularly focusing on tumor treatment and the tumor microenvironment, and features a seminal paper by Bai on aluminum nanoparticle-based immunotherapy. Cluster #1, “Red Tilapia,” suggests a niche in veterinary immunology with 112 members, while Cluster #2, "Tumor Cell," with 83 members, showcases personalized immunotherapy approaches. Protamine nanocapsules are at the core of Cluster #3, which explores delivery mechanisms for dengue vaccines, and Cluster #4 furthers the conversation on cancer nanovaccines, particularly against SARS-CoV-2. Cluster #5 focuses on dendritic cells’ role in nanoparticle vaccines, reflecting advances in vaccine immunogenicity. Novel delivery systems are the highlight of Cluster #6, showcasing innovative anti-nicotine vaccine delivery research. Cluster #7 zooms in on targeting immune cells with amphiphilic polyanhydride nanoparticles. Mesoporous silica nanoparticles for controlled drug delivery are discussed in Cluster #8, demonstrating the cross-disciplinary nature of nanotechnology. Gold nanoparticles, in Cluster #9, emerge as a promising tool for robust immunization strategies. Cluster #12 focuses on the characterization of nanoparticles, a crucial step for clinical translation, and Cluster #13 spotlights farinae-chitosan vaccines for asthma, indicating a focus on allergen-specific immunotherapy. These clusters, with their high silhouette values and distinctive keywords, reveal the evolving complexity and collaborative efforts within the nanovaccine research community.

Keywords With Strongest Citation Bursts

**Figure 9:**
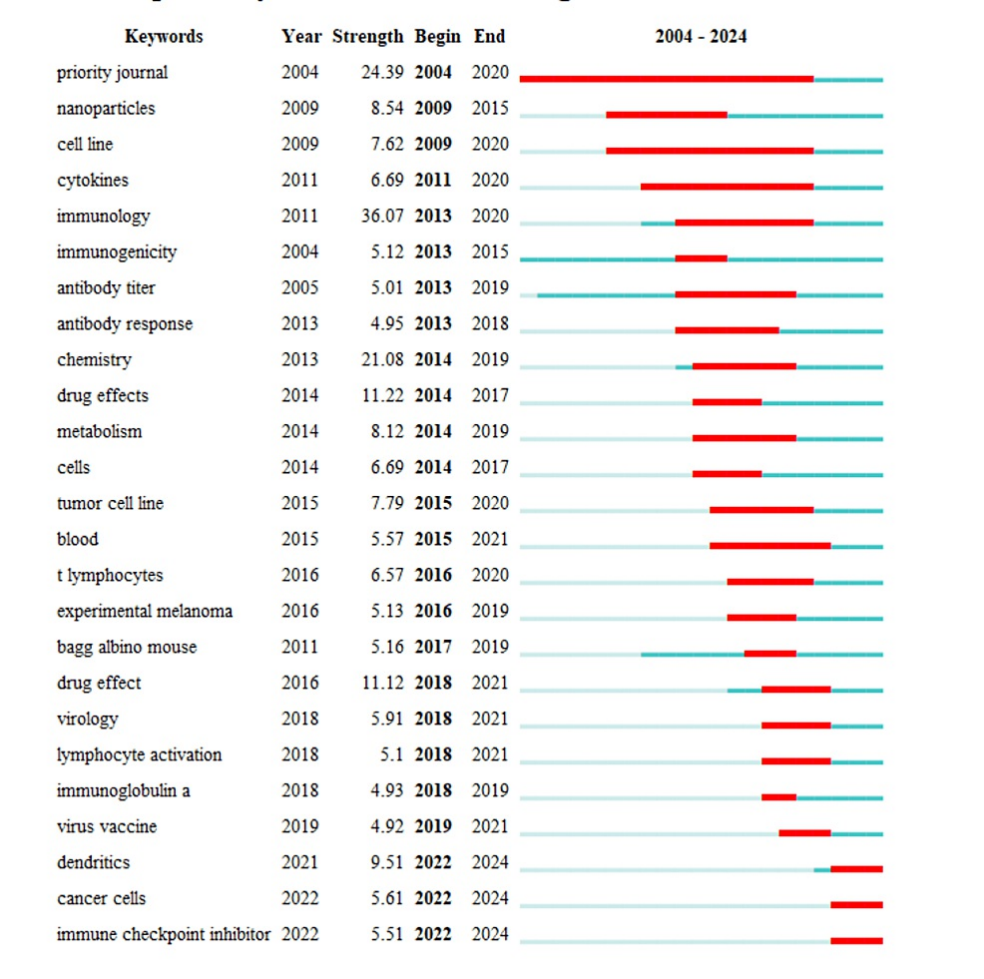
Keywords with strongest citation bursts

Figure 9 presents keywords with the strongest citation bursts, offering the intensity and timing of scientific interest in key topics, revealing shifts in the research landscape from 2004 to 2024. The sustained burst in “Priority Journal” citations from 2004 to 2020 underscores a consistent focus on high-impact research publications. The spike in “Nanoparticles” from 2009 to 2015 coincides with significant advancements in nanotechnology applications. Persistent interest in “Cell Line” and “Cytokines” up to 2020 reflects their importance in understanding cellular behavior and immune responses. A decade-long burst in “Immunology” aligns with the development of innovative vaccines and immunotherapies. Earlier peaks in “Immunogenicity” and “Antibody Titer” highlight a focus on vaccine response and effectiveness. “Antibody Response,” “Chemistry,” “Drug Effects,” and “Metabolism” each had periods of intense research, likely related to disease treatment innovations and biochemical studies. Keywords like “Cells,” “Tumor Cell Line,” “Blood,” “T Lymphocytes,” “Experimental Melanoma,” and “Bagg Albino Mouse” emphasize the role of cellular and animal models in health and disease research. Recent research bursts in “Dendritics,” “Cancer Cells,” and “Immune Checkpoint Inhibitor” from 2021 to 2024 suggest cutting-edge exploration in cancer therapy and immune modulation. The timeline indicates a responsive research community where emerging challenges and technological breakthroughs shape scholarly focus.

Research gaps and practical implications

In the realm of nanovaccine research, a thorough analysis of trend topics and thematic maps uncovers distinct research gaps. There is an opportunity to diversify the spectrum of pathogen-specific nanovaccines, extending beyond well-studied pathogens like “Streptococcus iniae” and “Helicobacter pylori” to encompass a broader range of infectious agents. Moreover, chronic diseases and noninfectious conditions such as autoimmune disorders and allergies remain relatively unexplored territories where nanovaccine applications could offer new therapeutic avenues. Research on niche diseases, including “papillomavirus infections,” reveals a need for tailored nanovaccine solutions targeting less prevalent yet significant health issues. The scarcity of longitudinal studies indicates a gap in our understanding of nanovaccines’ long-term efficacy and safety, a critical factor for their clinical success. Additionally, the concept of personalized nanovaccines, while hinted at through studies on immune molecules like tumor necrosis factors, has yet to become a mainstay in the research landscape.

From a practical perspective, the research has far-reaching implications, most especially in the field of cancer immunotherapy. The prioritization of dendritic and cancer cells suggests that nanovaccines can significantly disrupt treatment modes. The use of biodegradable materials, such as “polyanhydrides,” “lactic acid,” and “polyglycolic acid,” emphasizes the potential of delivery systems that can ensure patient safety and promote adherence. The eradication of radical diseases such as the “dengue virus” and “Yersinia pestis” is equally more visible, pointing to a future in which nanovaccines play a significant role in ending public health crises. Motor themes, such as “Streptococcus pneumoniae,” are prioritized and invested in, as evidenced by research funding channels. In this regard, there is a fluctuating amount of knowledge about animals, suggesting that animal models of research are being reassessed for relevance. In countries such as the United States, Sweden, and Australia, foundational themes are prioritized. It appears that the research will be integrated into human biology, population, and society to enhance vaccine access and articulate public health policy. If these gaps are properly addressed, the benefits will be vast. It will fast-track nanovaccine research and bridge the gap for incredible treatment options for diverse diseases and population age groups.

The bibliometric analysis using only Scopus and focusing on quantitative data comes with certain limitations. Scopus might not capture all relevant studies, particularly from lesser-known journals or in languages other than English, which could leave gaps in the analysis. Also, emphasizing metrics like citation counts could overshadow newer or less recognized research areas. Additionally, this quantitative focus overlooks the qualitative aspects of research, which are essential for a deeper and more nuanced understanding of any field. Including more qualitative analysis could provide vital insights into the context, motivations, and theoretical contributions that quantitative data alone cannot offer.

## Conclusions

This bibliometric analysis of nanovaccine research from 2004 to 2024 captures the field’s vibrant and dynamic nature, showcasing significant growth fueled by technological innovations and interdisciplinary collaboration. The field has seen a steady increase in publication trends, highlighting its transformative potential in vaccine development. Despite this progress, there is a notable decline in research focused on nanovaccines for rare and chronic diseases, pointing to critical gaps that need addressing. The study underscores the key contributions from leading countries and institutions, which reflect robust collaborative efforts across various domains, including cancer vaccines and novel delivery systems. However, challenges such as the long-term efficacy and safety of nanovaccines and the need for a broader spectrum of pathogen coverage and applications in chronic diseases remain. Future research should prioritize personalized nanovaccines and the development of safe, innovative delivery methods to enhance patient compliance and address public health needs. Addressing the identified research gaps will be essential for harnessing the full potential of nanovaccines in public health, particularly in preventing and treating diseases with significant public health impacts. The ongoing investment in this field is poised to yield substantial public health benefits, thanks to targeted delivery and biodegradable options that align with public health priorities.
